# Gorham-Stout syndrome of the shoulder

**DOI:** 10.1051/sicotj/2016015

**Published:** 2016-05-16

**Authors:** Ulrich Brunner, Kilian Rückl, Christian Konrads, Maximilian Rudert, Piet Plumhoff

**Affiliations:** 1 Department of Orthopaedic Surgery, Koenig-Ludwig Haus, Julius-Maximilians University Wuerzburg Brettreichstr. 11 97074 Wuerzburg Germany; 2 Department of Trauma, Shoulder and Hand Surgery, Hospital Agatharied Norbert-Kerkel-Platz 83734 Hausham Germany

**Keywords:** Rapid progression arthritis, Idiopathic osteolysis, Shoulder arthroplasty, Joint replacement, Vanishing bone disease

## Abstract

*Introduction*: Gorham-Stout syndrome (GSS) is a rare but severe subtype of idiopathic osteolysis. There are no guidelines for the treatment of GSS. We analysed different diagnostic and therapeutic regimes and we describe the sucessful treatment of GSS considering individual patient factors.

*Methods*: We diagnosed three patients with shoulder-specific GSS using clinical, radiological and histopathological examinations. Two out of three patients with similar clinical appearances were treated non-operatively. One patient was treated by reverse shoulder arthroplasty. All patients were analysed retrospectively using clinical and radiological evaluation with a mean follow-up of 42 (range 30–50) months.

*Results*: Two patients had few symptoms of GSS and were treated conservatively. One patient underwent arthroplasty, with a good clinical result. No additional therapy, such as radiation or anti-resorptive medications, was needed for the stable fixation of the prosthesis and the termination of osteolysis. In all patients we found good clinical outcomes with high patient satisfaction.

*Discussion*: GSS is diagnosed after exclusion of infectious, malignant, and systemic disorders. The diagnosis should be supported by clinical, radiological, and histopathological characteristics of patients. Different humoral and cellular changes have been reported in GSS, but lack sufficient supporting evidence. GSS is associated with angiomatous and lymphatic malformations. The changes in GSS and the theories of its pathophysiology may reveal.

## Introduction

Gorham-Stout syndrome (GSS) is a very rare, not curable disease [1]. Worldwide, only 200 cases are known counting all affected regions [2]. The pathogenesis is still unknown. GSS leads to massive restrictions in the quality of life. The overall mortality in patients with GSS is about 13% [3]. A systematic gathering of experiences with patients suffering GSS is essential for a better understanding of the disease and the development of effective therapies. This work analyses different courses and treatment options.

## Patients and methods

### Patient 1

**Figure 1.**
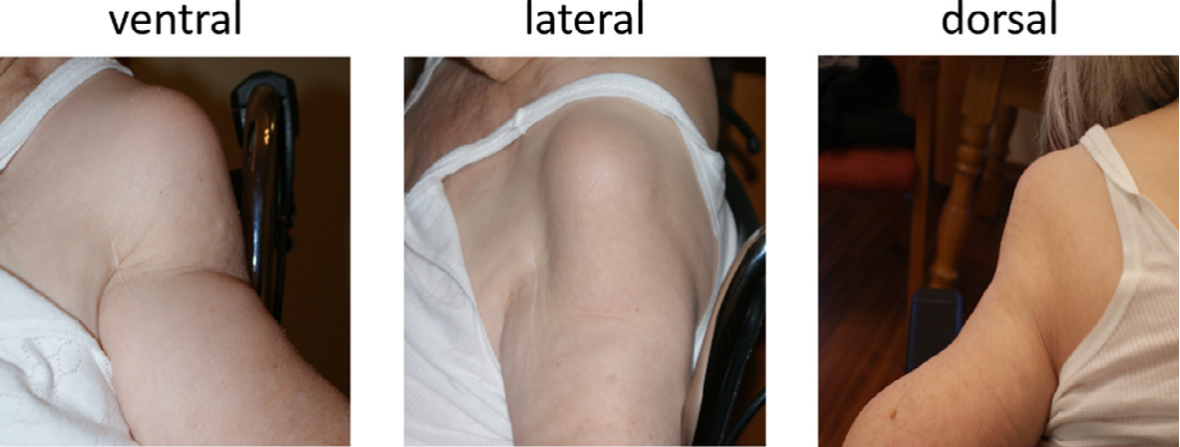
Left shoulder photographs of an 84-year-old female (patient 1) suffering Gorham-Stout syndrome in April 2011: the original contour of the shoulder vanished completely.

An 84-year-old female presented in our emergency department suffering pain, diffuse swelling and haematoma of the left upper arm (Figure 1) without history of a trauma.

**Figure 2.**
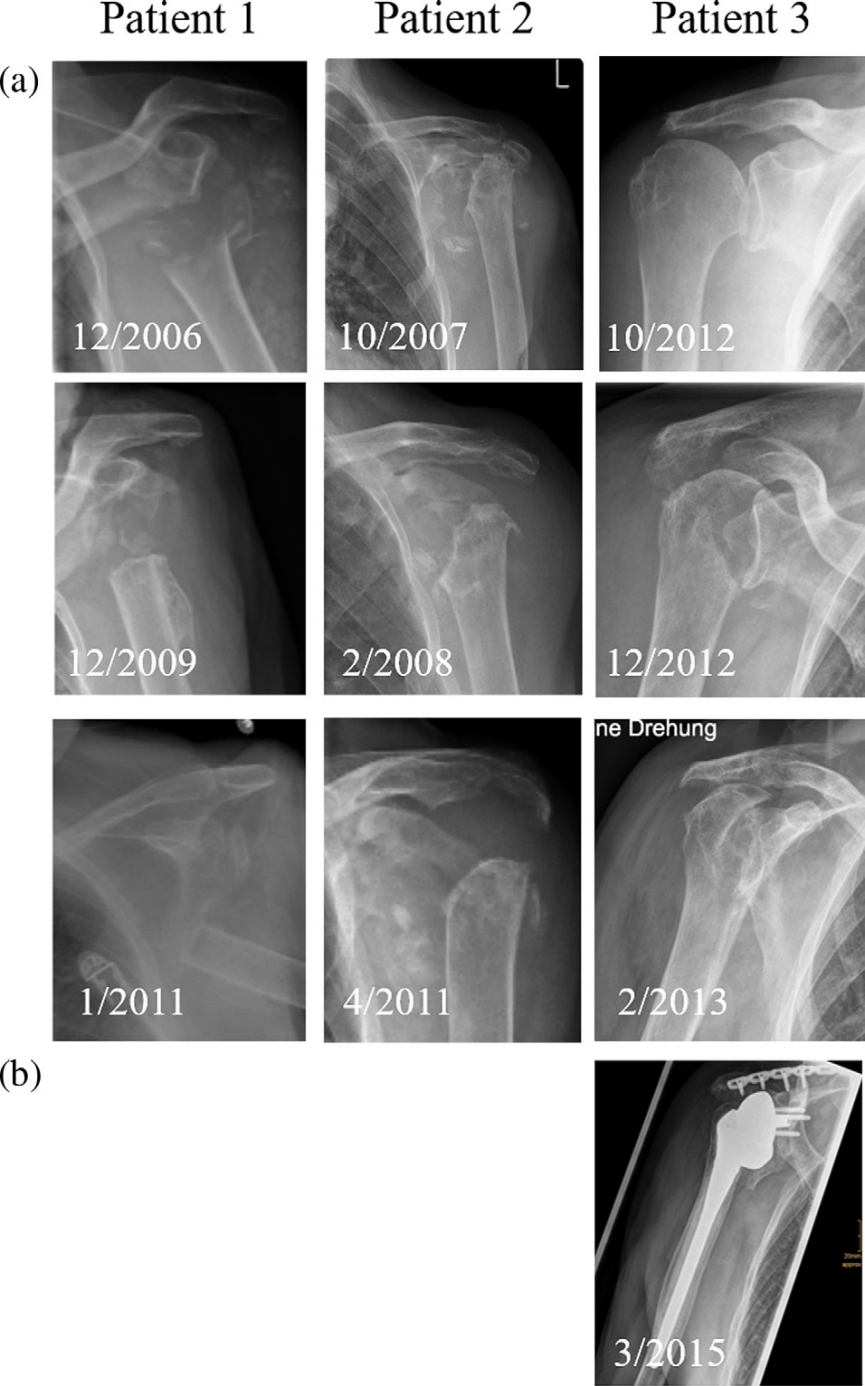
Antero-posterior radiographs of the involved shoulder of each of the three patients over time: (a) progressive osteolysis in all three patients; (b) implanted reverse shoulder prosthesis without loosening 12 months postoperatively.

**Figure 3.**
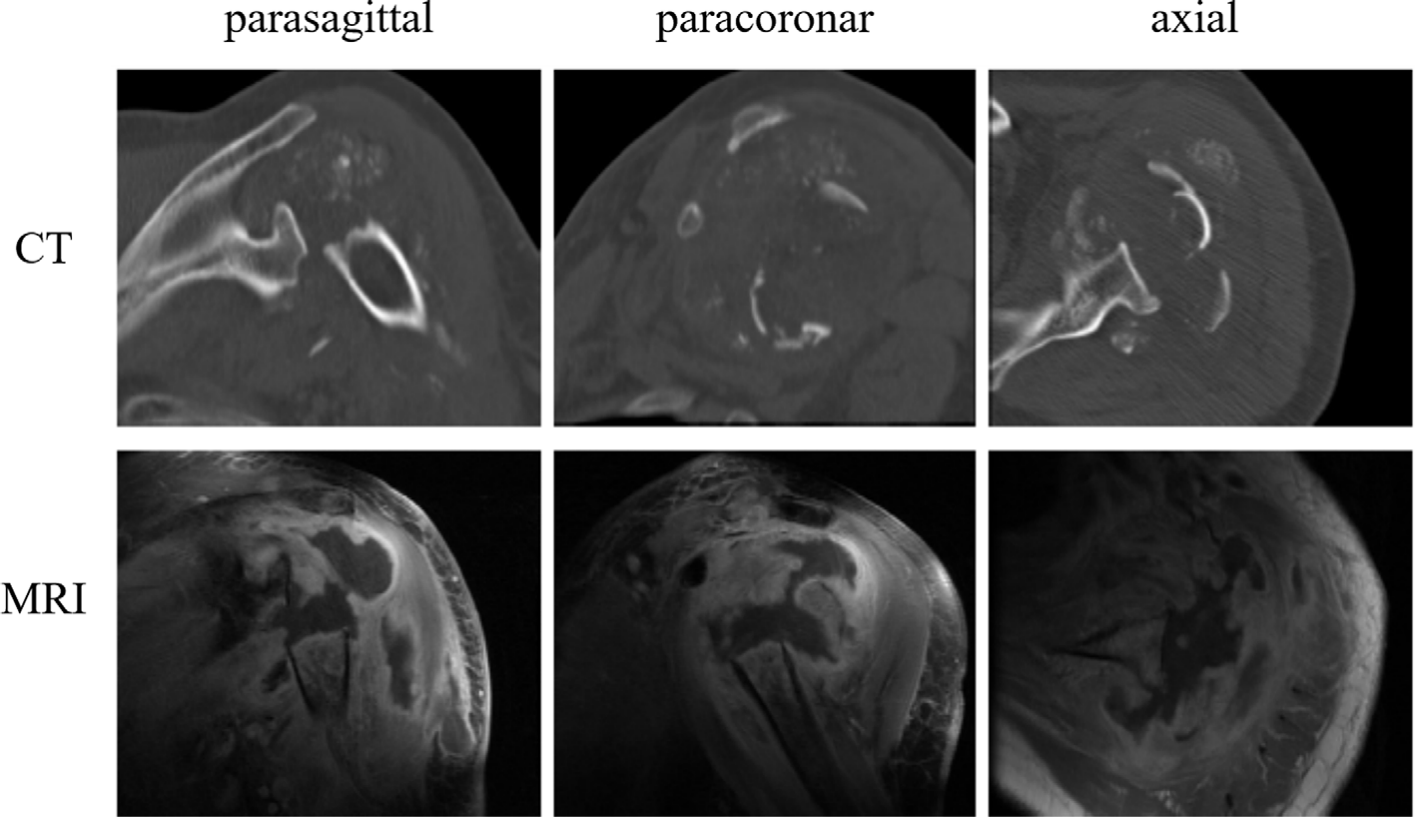
CT and MRI of the left shoulder (patient 1): massive osteolysis and tumour. Multiple similar calcifications were found in all three patients in CTs.

**Figure 4.**
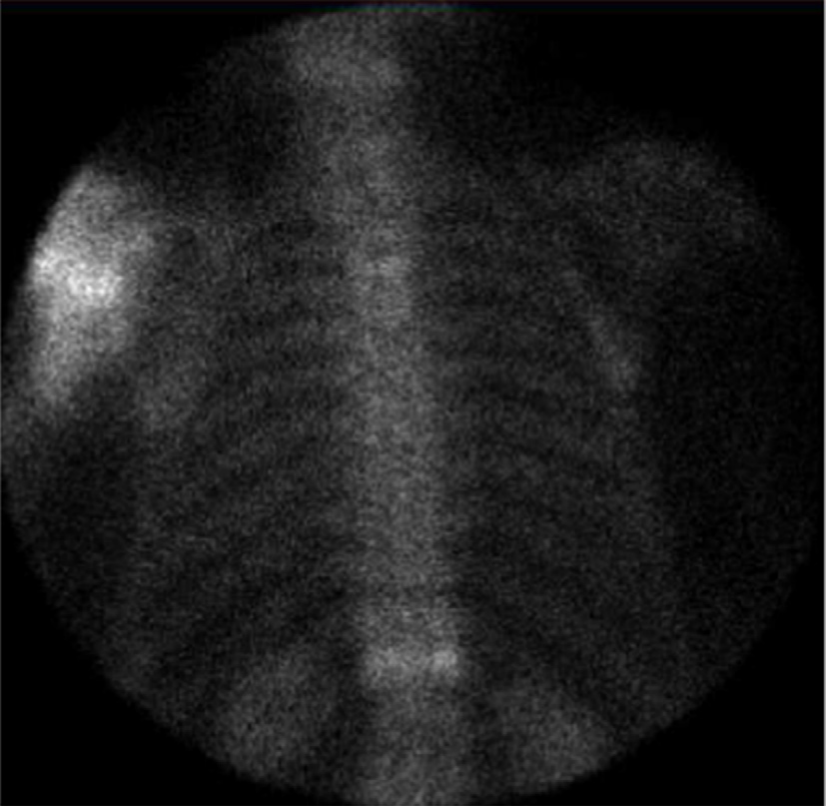
Bone scan (patient 1): excessive accumulation in the mineralization phase in the left shoulder.

Clinical chemistry showed slightly elevated inflammation parameters. Arthrocentesis did not show any signs of infection. Radiographs of the shoulder in three planes (Figure 2) and computer tomography (CT; Figure 3) demonstrated an extensive destruction of the humeral head and glenoid with bony fragmentation and massive calcification of the joint space. Arthroscopic biopsy with histopathological analysis confirmed regressive changes of the bone with signs of a chronic-inflammatory process. The material was sent to the reference centre for osteoradiology in Bremen, Germany, that also negated malignancy and presumed a neurogenic arthropathy. The scintigram attested an inconspicuous reperfusion phase but a slight accumulation in the soft tissue phase and an excessive accumulation in the mineralization phase (Figure 4).

Infection parameters declined in the course and a slight elevation of C-reactive protein (CRP) was likely to be caused by haematoma. We discussed the possibilities of prosthetic replacement but declined due to general health issues as well as reduced functional demand of the patient. After clinical convalescence, the patient went into ambulatory care.

Clinical and radiographic follow-up examinations were conducted every 12 months until four years of follow-up following initial presentation.

### Patient 2

A 92-year-old female patient presented after a low-impact contusion of the left shoulder with haematoma, but little pain. She showed a progressive restriction of active shoulder range-of-motion (ROM). Clinical examination showed an unstable shoulder joint, only fixed by soft tissues. Except for paraesthesia on D4 und D5, no neurological symptoms were found. The global muscular strength of the right arm was significantly reduced. Instability did not allow specific examination. Radiographs showed a progressive osteolysis of the left humeral head (Figure 2).

We assumed a neuropathic arthropathy of the left shoulder. The following magnetic resonance imaging (MRI) confirmed an advanced destruction of the humeral head and the glenoid with a large, fluid-filled central cavity and adjacent soft tissue structures with inflammatory enhancement. The clinical chemistry parameters and neurological examinations of the cervical spine were normal. Arthroscopic biopsy of the joint capsule with the following histopathological analysis revealed a high-grade reactive synovitis containing fibrosis, thickened fibrin, massive proliferation of lining cells and incorporated bone fragments.

The synopsis of these findings underlines the diagnosis of idiopathic osteolysis, GSS. Due to the reduced general health of the patient and the uncertain permanent fixation in vanishing bone, we desisted from a prosthetic reconstruction. Currently the patient is under successful conservative treatment regime with physiotherapy and pain management.

Clinical and radiographic follow-up examinations were conducted every three months until four years of follow-up after initial presentation.

### Patient 3

A 77-year-old female presented in our emergency department in December 2012 suffering from pain in the right shoulder. The right arm mobility was severely impaired. No history of trauma could be ascertained. At the time of presentation, a series of standard radiographs had already been obtained. Radiographs demonstrated a quickly progressing osteolysis of the humeral head and a pathological fracture of the right spina scapulae (Figure 2). In addition, we noticed suspicious changes in the bone as well as the soft tissues.

Several biopsies were taken (scapula, proximal humerus, glenoid) to establish a histologic diagnosis. The histological and microbiological analysis illustrated no evidence of an infection or malignancy. We assumed GSS. To exclude false negative results of the biopsy we planned histopathological examination of the remaining humeral head. We performed a plate fixation of the scapula, a resection of the humeral head, and we implanted a temporary articulating cement-spacer. Histopathology showed no signs of malignancy. We extended diagnostics using *620 MBq Tc-99m Oxidronacid*-Scintigram demonstrating no bone abnormalities other than hyperaemia and increased activity of osteoblasts of the proximal humerus. DOTATATE-PET-CT, MRI of the head, and general examination showed no pathological findings.

In the course, the patient needed an operation for a mitral valve abnormality. This delayed further treatment of the shoulder. Eleven months later, after recovering from the operation and with a negative microbiological result of a preoperative arthrocentesis, we replaced the cement-spacer using a reverse shoulder arthroplasty (RSA) (Aequalis, Tornier Surgical Implants^®^). Intraoperative swabs remained negative. The patient recovered without complications.

At the last follow-up, 12 months after RSA, the patient was very satisfied with only few restrictions in physically stressful activities. Radiographs showed no signs of prosthetic loosening or progressive osteolysis (Figure 2).

## Results and discussion

We treated three patients suffering GSS. Before establishing the diagnosis, we excluded possible differential diagnoses. In each patient, malignancy and infection was excluded. Radiological imaging showed typical progressive osteolysis without osteoplastic effects. Visceral comorbidities or ulcerative processes were not found. There was no specific pathology of angiomatous dysplasia and proliferation. All histopathological findings showed signs of chronic inflammation, which include angiogenesis. Reference centres presumed a neurogenetic arthropathy. Table 1 summarizes diagnostic findings in the three patients according to typical findings in GSS. All patients were satisfied with low pain levels according to the visual analogue pain scale at last follow-up examinations: patient one had 2/10, patient two 1/10 and patient three 1/10.

**Table 1.** Diagnostic results and treatment regimes of the three patients suffering Gorham-Stout disease.

Diagnostics	Patient 1	Patient 2	Patient 3
X-ray	Progressive osteolysis		
	No osteoplastic reaction		
CT/MRI	Progressive osteolysis		
	No osteoplastic reaction		
Histopathology	Regressive changes of the bone with findings of a chronic-inflammatory process	High-grade reactive synovitis with fibrosis, thickened fibrin, massive proliferation of lining cells, and incorporated bone fragments	No evidence of an active infection or malignancy
	No cell atypia		
General inspection	No hint of visceral concomitant disease		
Microbiology	Negative, no infection		
Treatment	Conservative	Conservative	Reverse shoulder arthroplasty

In 1955, Gorham first described 24 cases of a progressive osteolysis of one or more bones. Case histories typically only revealed minor trauma and patients often suffered from a pathological fracture additional to vascular malformation. Based on Gorham’s findings, a subtype of idiopathic osteolysis was named after him [1, 4].

Idiopathic osteolysis represents a very heterogeneous group of disorders with various clinical manifestations. Lots of them show genetic inheritability. Cases differ in severity of renal affection, prognosis and other factors. Hardegger et al. gave a concise overview (Table 2).

**Table 2.** Classification of “Idiopathic osteolysis” according to Hardegger et al. [5]. Patients with Gorham-Stout syndrome do not show renal involvement or systemic symptoms.

Type		Typical age of manifestation	Location of manifestation	Nephropathy	Prognosis
1	Hereditary multicentric osteolysis with dominant inheritance	Juvenile	Carpotarsal osteolysis, less often with affection of the radius and ulna	No	Good, disease arrest in adolescence
2	Hereditary multicentric osteolysis with recessive transmission	Juvenile	According to type 1, in addition severe generalized osteoporosis	No	Good, disease arrest in adolescence
3	Non hereditary multicentric osteolysis with nephropathy	Juvenile	Mainly carpometacarpal, rare tarsal affection, malignant hypertension	Yes, proteinuria in progressive renal pathology	Often unfavourable
4	Gorham-Stout syndrome	Age-independent	Typical: shoulder girdle, pelvis, facial skull	No	Mostly good. When in spine or chylothorax: lethality up to 50%
5	Winchester syndrome (hereditary, autosomal recessive)	Juvenile	Carpotarsal osteolysis and contractures, short stature, osteoporosis, corneal deterioration	No	Progressive

GSS is characterized as a progressive osteolysis of one or more bones or joints associated with functional deficits and pain [2]. Onset is age-independent and the patient’s age at time of diagnosis varied between one month and 75 years [5, 6]. Osteolysis can involve any bone but is most common in the shoulder girdle, pelvis, and facial skull; each represented by approximately 20% of all distributed cases [7]. Primary manifestation in the spine is seen less often; it rather occurs as secondary manifestation with 28 cases being reported [6].

We analysed three patients with isolated manifestation of GSS in the shoulder. In general, about 16% of patients with GSS show osteolysis of the shoulder girdle with 7.4% starting in the humerus [3]. Isolated manifestations of GSS in this region are rarely reported. Only seven publications are listed in the database “PubMed” searching for “shoulder” and “Gorham-Stout” in combination [6, 8–13]. Table 3 lists the articles about shoulder-specific manifestations of GSS. One article was excluded as the shoulder was not the only location of manifestation [6].

**Table 3.** Overview of the PubMed listed shoulder-specific case-reports of Gorham-Stout syndrome using the following search: “Gorham” and “shoulder”.

Paper	Patient	Comorbidity	Treatment
Buerfeind A., 2010, Orthopäde	24y, m	None or not reported	Reverse shoulder arthroplasty
Hugo B., 1989, RoFo	49y, f	None or not reported	No specific therapy
Busilacchi A., 2012, JSES	36y, m	None or not reported	Non-cemented arthroplasty (Lima)
Pans S., 1999, JSES	72y, m	Orchiectomy due to tuberculosis, polycystic renal disease	Biopsy, arthroscopy because of suspected infection. Radiation with 40 Gy in 20 units in two months. Arthroscopic debridement. No progression.
Garbers E., 2011, Case Rep Rheumatology	77y, f	Chronic bronchial asthma, hyperthyroidism, TIA, manifest osteoporosis	Anatomic TSA (Affinis, Mathys) of right side, conservative treatment of left side; 1-y-follow-up without loosening
Hofbauer, L.C., 1999, Rheumatology (Oxford)	8y, m	None	No radiation because of patient’s age

TSA = Total shoulder arthroplasty; TIA = transitory ischemic attack.

Due to the rarity and the heterogeneous appearance, GSS should stay a diagnosis of exclusion [14]. Before determining the diagnosis, a wide range of differential diagnoses is to be considered. Among others, this consists of lymphangioma, angiosarcoma, essential or hereditary osteolysis, as well as bony manifestations of systemic diseases, such as rheumatoid arthritis, syphilis, complex regional pain syndrome (CRPS), gout, psoriatic arthritis, aseptic necrosis, and hyperparathyroidism [7, 15].

To establish the diagnosis of GSS, the following eight histopathological and clinical findings should be fulfilled [3, 16]: (1) radiographic detection of osteolysis; (2) exclusion of cellular atypia; (3) absence of osteoplastic reaction; (4) detection of a local progressive growing lesion; (5) exclusion of an ulcerating growing lesion; (6) exclusion of a visceral concomitant disease; (7) positive histological proof of angiomatous dysplasia and proliferation; (8) exclusion of a hereditary, metabolic, neoplastic, immunologic or infectious aetiology.

Each of the three patients reported in this paper fulfilled all eight diagnostic attributes. In each patient, malignancy and infection were ruled out. Radiographic imaging showed typical progressive osteolysis without osteoplastic effects. Visceral co-morbidities or ulcerative processes were not found. There was no angiomatous dysplasia and proliferation, but there were signs of chronic inflammation with significant angiogenesis. Various histopathological reference centres presumed a neurogenetic arthropathy (Table 1).

### Pathogenesis

Histological proof of angiomatous dysplasia and proliferation is characteristic for GSS [16]. Until the beginning of 2016, pathogenesis is still unknown while various changes in molecular mechanisms were reported.

Immunohistochemical testing proved an overexpression of *cluster of differentiation-105* (*CD105*)*/Endoglin* on vascular endothelial cells (compared to samples of osseous haemangiomas), as it occurs in areas of active bone growth [17]. This over-expression could cause the observed excessive neovascularization.

Other research groups emphasized an increased concentration of *plate-derived growth factor-BB* (*PDGF-BB*), a subtype of *PDGF* [18]. The family of *PDGFs* plays an important role in pathogenesis of lymphedema-distichiasis as well as lymphangiogenesis in tumours. In a similar manner, *PDGF-BB*-triggered pathway could play a crucial role in the pathogenesis of GSS. This theory was supported based on observations of a patient who concomitantly showed a massive lymphangiomatous malformation of the lower extremity [19].

Another approach suggests that osteoclast activation by *Interleukin-6* (*IL-6*) plays a crucial role in GSS. Devlin et al. showed an increased *IL-6* concentration in patients with GSS [20]. *IL-6* stimulates osteoclasts, which can lead to a massive bone resorption. Monocyte cultures showed an increase in osteoclast differentiation and bone resorption after adding serum of GSS patients. In summary, *IL-6* triggers an increased sensitivity of precursor cells to humoral factors (*receptor activator of nuclear factor kappa-B ligand* (*RANKL*) and *macrophage colony-stimulating factor* (*M-CSF*)). This results in the activation and differentiation of osteoclasts leading to the characteristic osteolysis [21].

Colucci et al. isolated a homologous monocytic cell population from a patient with GSS and found characteristics of immature osteoclasts. These cells produce angiogenic factors (*vascular endothelial growth factor A* (*VEGF-A*) and *IL-8*) and osteoclastogenetic factors (*IL-1 beta, IL-6, TGF* (*transforming growth factor*)*-beta 1*) [22]. In the presence of inflammatory mediators, these cells also showed the ability to migrate and digest extracellular matrix through expression of metallomatrix proteins.

**Figure 5.**
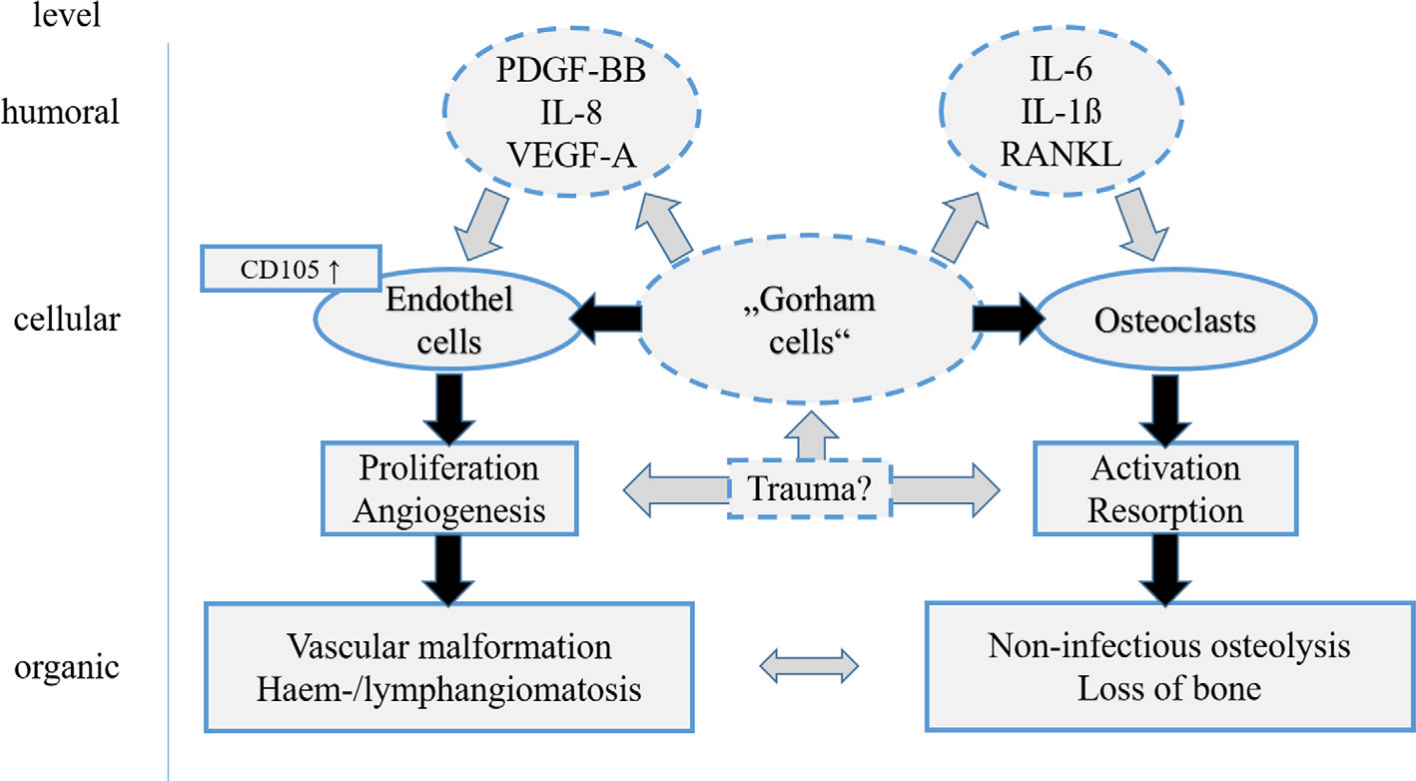
Overview of the existing theories regarding pathomechanism in Gorham-Stout syndrome. Broken lines and grey arrows show theoretical considerations still lacking proof. Role of trauma still remains uncertain. Elevated levels of *Il-6* and differentiation factors *Il-1β* and *RANKL* may stimulate osteoclasts and lead to bone resorption while *PDGF-BB, IL-8*, and *VEGF-A* may activate endothelial cells and others via *CD105* and *TGF-beta1-receptor*. This may result in vascular malformation and lymphangiomatosis. Both are characteristics of Gorham-Stout syndrome.

Based on these observations Seefried et al. examined possible causal connections. Therefore, they included both the cellular level observed by Colucci’s “Gorham-cells” and the humoral changes with *IL-6* increase and angiogenic factors [23]. Figure 5 shows possible interactions between humoral and cellular mechanisms during pathogenesis of GSS.

### Clinical course and therapeutic options

The three patients reported in this paper received individualized treatments mainly due to differences in general health conditions, comorbidities, and function claims.

Publications describing new therapeutic options report partial regression up to total remission of osteolysis. In addition to positive effects of radiation, successful use of interferon alpha-2b in combination with bisphosphonates was described [2, 10, 24].

Two patients maintained conservative treatment. Both were old (84 and 92 years) and of reduced general health. We avoided prosthetic replacement. The patients only occasionally complained of mild pain sensations caused by bone crepitation. We see no benefit in applying radiation as long as osteolysis remains local and progression is slow. Chronic renal failure did not allow administration of bisphosphonates in these cases.

The 77-year-old female was in good general health. At first presentation, she had a fracture of the spina scapulae without a history of a trauma. Four months later, she suffered a total collapse of the humeral head (Figure 2). Intraarticular swabs stayed negative and the histological examination of a biopsy and the later resected remains of the humeral head did not show malignancy. After osteosynthesis of the scapular spine fracture, we inserted a cement-spacer for the glenohumeral joint. In a second operation, we implanted a RSA. This surgery was delayed by eight months due to a necessary reconstruction of the mitral defect. The bone stock was reduced especially at the lower rim of the glenoid. This is more likely due to the prolonged spacer wear than due to a primary osteolysis at this location. Thus, a stable fixation of the Metaglene was possible without bone graft. X-rays (Figure 2) showed limited loss of bone at the lower glenoid rim in similar appearance to the classic radiographic signs of notching. After one-year follow-up, there was not any progress of bone loss and no signs of loosening. We did not consider radiation therapy to be needed.

Out of six patients suffering shoulder-specific GSS reported in the literature, three patients were treated with arthroplasty but no author could provide long-term results. The remaining patients were treated with radiotherapy, operative debridement, or conservative regime [8–13].

In general, the clinical course of GSS varies widely, ranging from spontaneous remission, slow progressive osteolysis with some mild clinical symptoms to fatal outcome, usually in the context of a chylothorax [4, 6]. Pathogenically, the direct migration of dysplastic lymphocytic vessels into the thorax or the erosion of the thoracic duct is being discussed [24]. This also supports the theory of a lymphatic pathogenesis in GSS [19]. Pleurodesis, ligation of lymphatic vessels, radiotherapy, or interferon therapy presents viable therapeutic options [24, 25]. The surgical ligation of lymphatic vessels significantly improved lethality from 36% to 64% [26].

Another severe complication of GSS is spinal instability caused by osteolytic destruction of vertebrae. Here, early intervention is essential. The very high mortality, specified in the literature of 33–53%, also includes complications of the often coexisting chylothorax [3, 7]. In a literature review of 175 cases with GSS, Florchinger et al. quantified a mean mortality of 13.3% [3].

From an osteological point of view, a detailed evaluation of bone metabolism and supportive medication for bone growth might be important: e.g. vitamin K substitution and interferon alpha-2b or bisphosphonate therapy is advisable. The latter was not an option for our patients due to an advanced renal insufficiency. Until the beginning of 2016 the effect of bone-supportive medication in GSS still lacks evidence. Due to the administration in only few individuals, proof of efficiency is still lacking.

## Conclusions

This work presented three patients suffering from Gorham-Stout syndrome, an extremely rare form of idiopathic osteolysis. Little is known about this disease and therapeutic approaches are only empiric so far. The first manifestation occurs regardless of age and gender. The most involved regions are shoulder, pelvis, and facial skull. Prognosis is usually good. However, if the spine is affected or chylothorax develops, a lethality up to 50% was described.

Shoulder-specific manifestations of GSS were rarely reported. The three patients presented in this work suffered isolated GSS of the shoulder. Mainly because of individual differences in general health, comorbidities, and function demands, patients were treated with different therapeutic regimes. One patient was successfully treated with reverse shoulder arthroplasty. Two other patients received conservative treatment; also with satisfactory results. We did not prescribe radiotherapy or specific medication on any of these patients.

All currently existing pathologic theories follow very different approaches. Until a definitive molecular background of the disease can be identified, treatment of GSS must be based on empirical data. The exchange of experiences in diagnostics and treatment of this very rare disease has a high priority for improving the care of affected patients.

## Conflict of interest

All authors declare no conflict of interest in relation with this paper.
